# Comparative Transcriptome Analysis Reveals Regulatory Factors Involved in *Vibrio Parahaemolyticus* Biofilm Formation

**DOI:** 10.3389/fcimb.2022.917131

**Published:** 2022-07-11

**Authors:** Qiuyu Wang, Pengfei Wang, Pingping Liu, Jie Ou

**Affiliations:** ^1^ College of Food Science and Technology, Shanghai Ocean University, Shanghai, China; ^2^ Laboratory of Quality and Safety Risk Assessment for Aquatic Products on Storage and Preservation (Shanghai), Ministry of Agriculture and Rural Affairs, Shanghai, China; ^3^ Shanghai Engineering Research Center of Aquatic-Product Processing and Preservation, Shanghai, China

**Keywords:** *Vibrio parahaemolyticus*, biofilm, transcriptomics, two component system, quorum sensing

## Abstract

*Vibrio parahaemolyticus* biofilm poses a serious threat to food safety and human health. However, there is limited knowledge of transcriptional regulatory mechanism during the biofilm formation of this organism. Hence, the RNA sequencing technique was employed to compare the differences in transcriptome profiles between planktonic and biofilm state of *V. parahaemolyticus* ATCC33847 in this study. Collections of mRNA from planktonic and biofilm cells cultured at 25°C for 36 h were sequenced by studying their biological characteristics. The results showed that there were significant differences in the expression levels of 956 genes in biofilms compared with planktonic cells. These differences suggested that two-component regulatory system (TCS) and quorum sensing (QS) regulated *V. parahaemolyticus* biofilm formation by affecting important factors such as flagellar motility, Extracellular polymeric substance (EPS) secretion, tripartite ATP-independent (TRAP) transport system and ATP binding cassette (ABC) transport system. The present work in transcriptomics serves as a basis for future studies examining the complex network systems that regulate bacterial biofilm formation.

## Introduction

*V. parahaemolyticus* is a halophilic gram-negative foodborne pathogen that distributing a variety of seafood. Consumption of undercooked food contaminated *V. parahaemolyticus* can cause nausea, vomiting, diarrhea and other symptoms of gastroenteritis and food poisoning (Su and Liu, 2007). In serious cases, it may lead to sepsis and death (A.Broberg et al., 2011). Recent years, the frequent occurrence of food poisoning caused by *V. parahaemolyticus* has threatened public safety, and its severity has exceeded that of *Salmonella* food poisoning, seriously threatening people's health and causing huge losses of economic property. During food processing, *V. parahaemolyticus* contaminates equipment by forming biofilms on abiotic surfaces such as stainless steel, polyvinyl chloride and glass, and contaminates food by forming biofilms on biological surfaces such as aquatic products, causing cross-infection and showing significant antibiotic resistance (Wang et al., 2022).

In *Vibrio* species, biofilm formation involves many transcriptional regulators, especially two-component regulatory system and quorum sensing regulators (Wuichet et al., 2010). Prokaryotic cells utilize histidine (His) and aspartate (Asp) phosphorylation for signaling in various life activities, which is called two-component regulatory system (TCS). A typical two-component regulatory system consists of a sensor histidine kinase (HK) and a response regulatory protein (RR) (Kilmury and Burrows, 2018). QS is an intercellular communication process that relies on the level of extracellular signaling molecules (also known as autoinducers), allowing bacteria to share information about cell density and regulate their gene expression accordingly (Waters and Bassler, 2005). As the bacterial population density increases, autoinducers (AI) are synthesized and accumulated in the environment. Binding to the QS receptor results in changes in gene expression when the AI ​​concentration increases to a threshold (Papenfort and Bassler, 2016). The QS system is involved in the regulation of many bacterial life processes, such as bioluminescence, biofilm formation, secondary metabolite production, DNA uptake capacity, virulence factor secretion and biocorrosion (Ng and Bassler, 2009).

**Figure 1:**
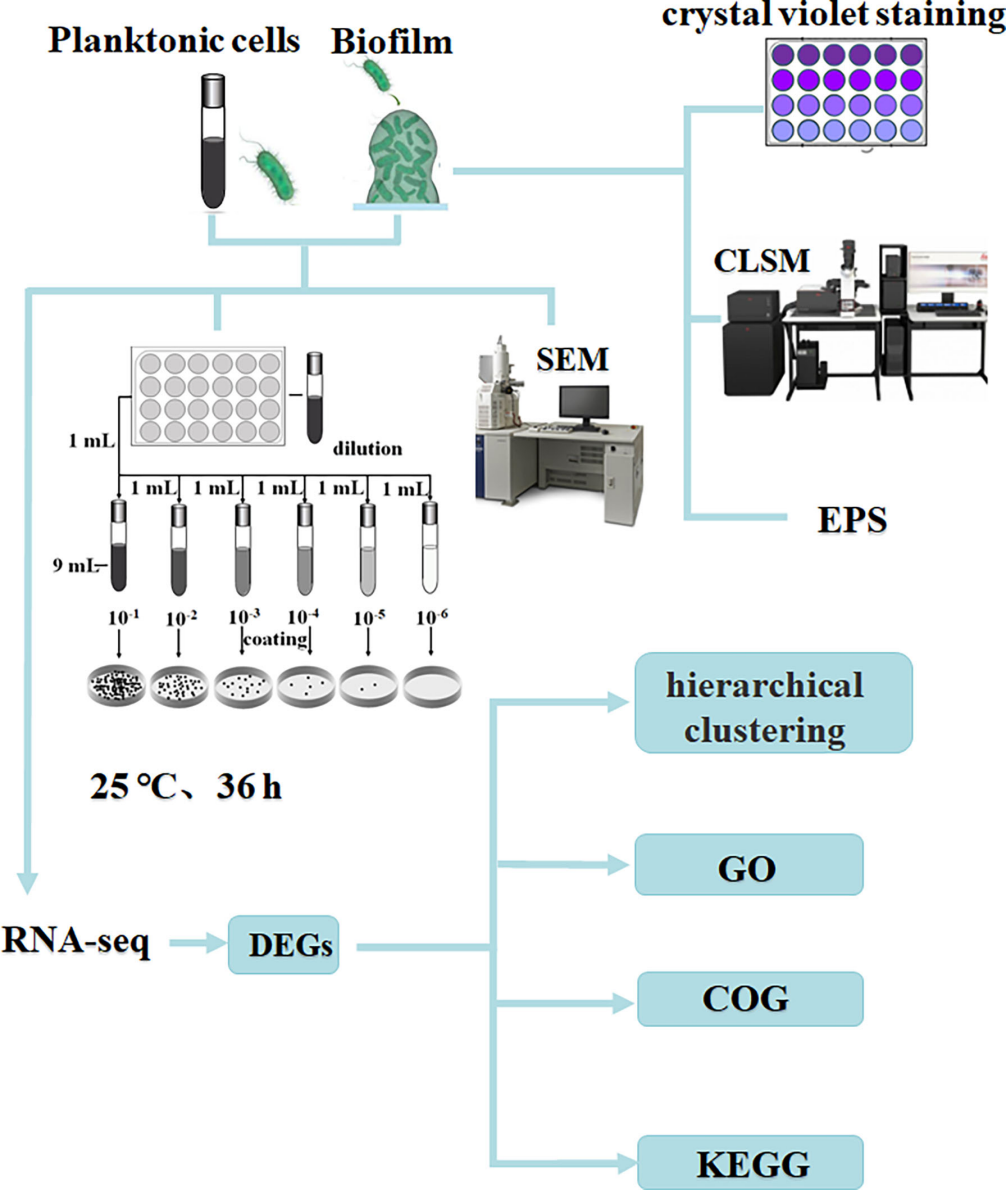
Experimental flow chart.

Methods to study biofilm formation based on transcriptomic analysis of gene expression differences between planktonic and biofilm cells have been widely used. For example, RD Waite et al. (Waite et al., 2005)found that *Pseudomonas aeruginosa* developed biofilms associated with planktonic cultures. Xu et al. found the role of *pmrA* in biofilm-associated cells (Xu et al., 2021). Rumbo-Feal et al. found amino acid and fatty acid metabolism, motility, active transport, DNA methylation, iron acquisition, transcriptional regulation and QS have undergone important changes in the process of biofilm formation (Rumbo-Feal et al., 2013). RNA sequencing using the information-rich Illumina system has developed into a sensitive and rapid technique for studying microbial transcriptional profiles (Marioni et al., 2008). At present, there are many studies on the regulation mechanism of *V. parahaemolyticus* biofilm formation, but there are few studies using the Illumina system to compare planktonic cells and biofilm formation, and the research on the regulation mechanism of biofilm formation is not comprehensive enough. In this study, the sequencing instrument Illumina NovaSeq 6000 was used to sequence planktonic and biofilm state of *V. parahaemolyticus*. The purpose of this study was to compare the gene expression differences between planktonic and biofilm cells using transcriptome sequencing technology, to gain insight into the regulatory mechanism of *V. parahaemolyticus* biofilm formation. The research process is shown in **Figure 1**.

## Materials and Methods

### Strain Activation and Cultivation

*V. parahaemolyticus* strain (stored in 75% glycerol at -80°C) was inoculated into 10 mL of tryptic soy broth (TSB) broth (3% NaCl), and cultured overnight at 37°C with constant shaking (180 r/min). The bacterial liquid was inoculated on a thiosulfate citrate bile salts sucrose agar (TCBS) plate for screening by streaking, and cultured upside down at 37°C for 12-18 h. Pick a suitable single colony into 10 mL TSB broth (3% NaCl), culture at 37°C, 180 r/min for 10-12 h, centrifuge at 4000 r/min for 10 min, wash the bacterial pellet with 0.85% normal saline three times, and diluted to *OD*_600_=0.6 (about 8 log CFU/mL), used as an inoculum for later use.

### Formation and Viability Determination of Planktonic and Biofilm Cells

To study the formation of biofilm bacteria at different times, 10 μL of the above bacterial solution and 990 μL of TSB (3% NaCl) were added to a 24-well plate, and the 24-well plate was placed in a well-sealed sterile homogeneous bag, cultured at 25°C for 6, 12, 18, 24, 30, 36, 42, 48, 54 and 60 h, respectively.

The bacterial solution was inoculated into TSB broth (3% NaCl) at a ratio of 1:99, a total of 10 mL, and cultured at 37°C with constant shaking (180 r/min) for 6, 12, 18, 24, 30, 36, 42, 48, 54, and 60 h, respectively.

The number of viable bacteria of planktonic and biofilm cells in different time periods was measured by plate counting method. The biofilm production was measured by crystal violet staining. Each well was gently washed 3 times with 1 × phosphate-buffered saline (PBS), dried in an open oven for 30 min, stained with 1 mL of 0.1% crystal violet for 30 min, and then and then solubilized in 1 mL 95% ethanol for 30 min. The optical density of each well was measured at 600 nm (Krom et al., 2007).

### Scanning Electron Microscopy

Due to its high-resolution imaging capability, SEM can directly observe changes in bacterial cell morphology. The planktonic bacteria and biofilm were cultured for 6 h, 18 h, 36 h and 60 h, and the planktonic bacteria were centrifuged at 10,000 r/min for 5 min. Planktonic bacteria and biofilms were subjected to gradient dehydration for 10 min with 30%, 50%, 70%, 90%, 100% and 100% ethanol. The dehydrated samples were air-dried on an ultra-clean bench, and then subjected to vacuum gold spraying. Finally, the samples were observed using a high-resolution benchtop SEM (Hu et al., 2018), with SEM magnifications ranging from 5.00 K× to 10.00 K×.

### Confocal Laser Scanning Microscopy

Place round glass slides in a 24-well plate, culture for 6 h, 18 h, 36 h and 60 h, remove planktonic bacteria, wash three times with 1 × PBS, and add 1 mL of 4% glutaraldehyde solution to fix at 4°C 30 min; stained with freshly prepared SYBR Green I dye for 30 min, protect from light to prevent fluorescence quenching; carefully remove the slide with tweezers, and use CLSM to acquire images after air-drying for 20 min (Beyenal et al., 2004).

### Extracellular Polymeric Substance Analysis

Biofilms were collected by scraping in 1 mL of 10 mM KCl, pretreated with sonication for 1 min (5 s operation, 5 s pause, 3.5 Hz), and the suspension was centrifuged at 4,000 r/min for 20 min at 4°C, and then filtered through a 0.22 μm membrane. Extracellular proteins were quantified by Lowry protein kit. Exopolysaccharides were quantified by the phenol-sulfuric acid method (Han-Shin and Hee-Deung, 2013).

### RNA Isolation, Library Construction and Sequencing

Planktonic and biofilm cells cultured at 25°C for 36 h were collected, rapidly lysed with Trizol reagent, reduced to powder under liquid nitrogen, and grind with a mortar and pestle (Rumbo-Feal et al., 2013). Bacterial total RNA was extracted and rRNA was removed, and the enriched mRNA was fragmented into short fragments using fragmentation buffer and reverse transcribed into cDNA using random primers, which were checked for quality. Illumina sequencing was performed when QC was completed and requirements were met. Sequencing was performed on the instrument Illumina NovaSeq 6000, and the SBS and cluster reagent cartridges were first thawed. For the NovaSeq Xp workflow, load ExAmp mix onto the flow cell and load the library tube into the thawed cluster cartridge. From the software interface, select Sequence and specify a single flow cell run. Then unload consumables from the previous run and load new consumables for the current run. Specify run parameters from the Run Setup screen. The run is monitored on the sequence screen after specifying the run parameters from the run setup screen, and the data is transferred to the specified output folder. After sequencing is complete, instrument cleaning starts automatically.

### Transcriptomic Data Processing

Raw RNA-sequencing data was filtered using Cutadapt (Martin, 2011) (v1.9.1) to remove contamination, adapters, and low-quality reads, and alignment of short reads using Bowtie2 (Bray et al., 2016) (v2.2.6) with *Vibrio parahaemolyticus* RIMD 2210633 as the reference genome. Make transcripts more comprehensive by discovering new transcripts with Rockhopper. Htseq (Anders et al., 2015) (v0.6.1) and FPKM (Fragments Per Kilo bases per Million reads) (Mortazavi et al., 2008) (Mortazavi et al.,2008) methods were used to calculate gene expression, and edgeR (Robinson et al., 2010) (v3.4.6) of the Bioconductor package was used to identify the difference in gene expression between samples with selection criteria of *q* value (fdr, padj) <= 0.05 and absolute log_2_ fold change (|log_2_ FC|) > 1. Gene Ontology (GO) (Harris et al., 2004) functional annotation was performed using GOseq (Young et al., 2010) (Young et al., 2010) to describe molecular functions, cellular components, and biological processes associated with biofilm expression. The Kyoto Encyclopedia of Genes and Genomes (KEGG) (Kanehisa and Goto, 2000) pathway enrichment analysis analyzed genes involved in different metabolic pathways. The function of the protein was predicted by the protein database COG (Cluster of Orthologous Groups of proteins) (Tatusov et al., 2000).

## Results

### Determination of the Number of Viable Cells of Planktonic and Biofilm Cells

**Figure 2:**
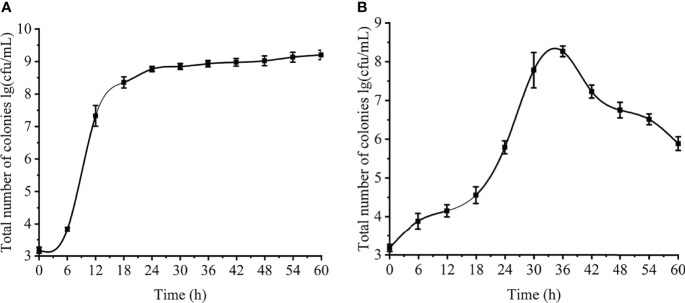
Changes of planktonic and biofilm cells at different time. **(A)** planktonic cells. **(B)** biofilm.

The changes in the number of viable cells of *V. parahaemolyticus* planktonic and biofilm cells were showed in **Figure 2**. The number of viable cells of planktonic cells gradually increased with the extension of the culture time, rapidly growing to about 8 log CFU/mL at 10-18 h, and tending to be stable to about 9 log CFU/mL at 23-24 h (**Figure 2A**). The change of the number of viable bacteria in the biofilm first increased at 0 to 36 h and then decreased at 36 to 60 h. The number of viable bacteria reached a maximum of about 8.4 log CFU/mL at 36 h, and slowly decreased to about 5.7 log CFU/mL at 60 h. (**Figure 2B**). The formation process of the biofilm was verified. First, it adhered and aggregated on the contact surface, and then continued to increase in value. The number of viable bacteria continued to increase until the environment was not suitable for growth, and the mature biofilm began to disperse. The changes of viable counts of planktonic and biofilm cells revealed the growth process of *V. parahaemolyticus* in different states.

### Biofilm Formation

**Figure 3:**
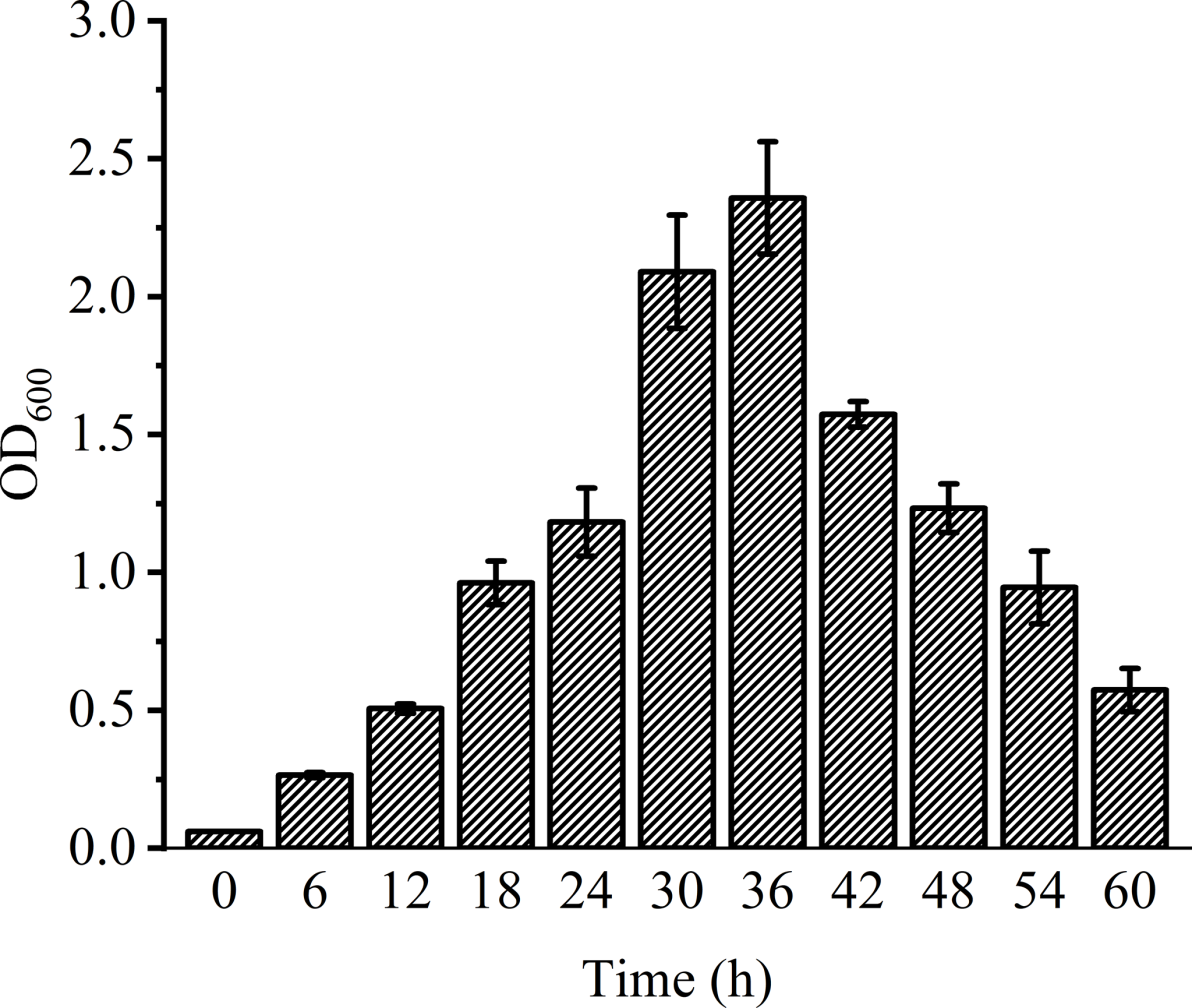
Biofilm formation of *V. parahaemolyticus*.

The amount of biofilm formation was observed by crystal violet staining. The results are shown in **Figure 3**. At 6-24 h, the biofilm grew slowly and steadily; at 24-36 h, when the bacteria just entered the mature stage, the biofilm grew rapidly, and increased to the maximum at about 36 h. (*OD*_600_=2.477); at 42-60 h, the amount of biofilm decreased sharply (*OD*_600_=0.483) and the mature biofilm was dispersed due to the bacteria still reproducing, the consumption of nutrients in the orifice plate and the accumulation of metabolic waste.

### Scanning Electron Microscopy

**Figure 4:**
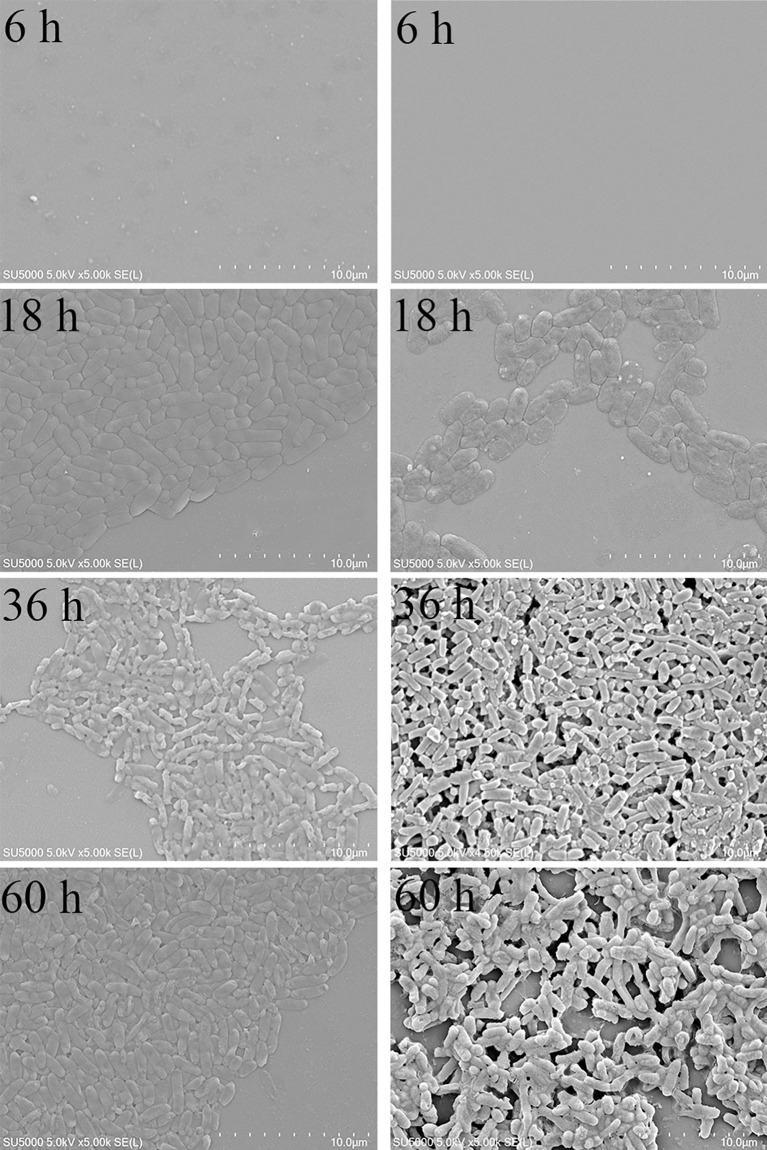
SEM results of planktonic and biofilm cells of *V. parahaemolyticus*. **(A)** planktonic cells. **(B)** biofilm.

The external morphology of planktonic bacteria (**Figure 4A**) and biofilm (**Figure 4B**) was observed by SEM. The number of planktonic bacteria increased gradually with the growth of time. At 6 h, no bacteria were found; at 18 h, the bacteria were in the logarithmic growth phase, and the cell morphology was typical at this time, and the arc-shaped and rod-shaped structures of the bacteria could be clearly seen, and the cell surface was smooth and round; 36 h and 60 h were both in the stable growth phase, the number of cells was not much different, and the cell morphology might change during this period, and a small amount of bacteria could be observed to appear autolysis. The number of bacteria in the biofilm gradually increased from 6 h to 36 h, and the number of bacteria in the biofilm decreased significantly at 60 h. At 36 h, the cells adhered more tightly, almost completely covering the contact surface, forming a tight whole. The extracellular polymers produced by bacteria help the cells to stack together and form a three-dimensional structure with a certain thickness. At 60 h, the adhesion strength was significantly reduced, the blank area of ​​the contact surface increased significantly, and the biofilm was damaged, indicating that the mature biofilm was dispersed and individual cells fell off. The growth process of planktonic and biofilm cells of *V. parahaemolyticus* is quite different.

### Confocal Laser Scanning Microscopy

**Figure 5:**
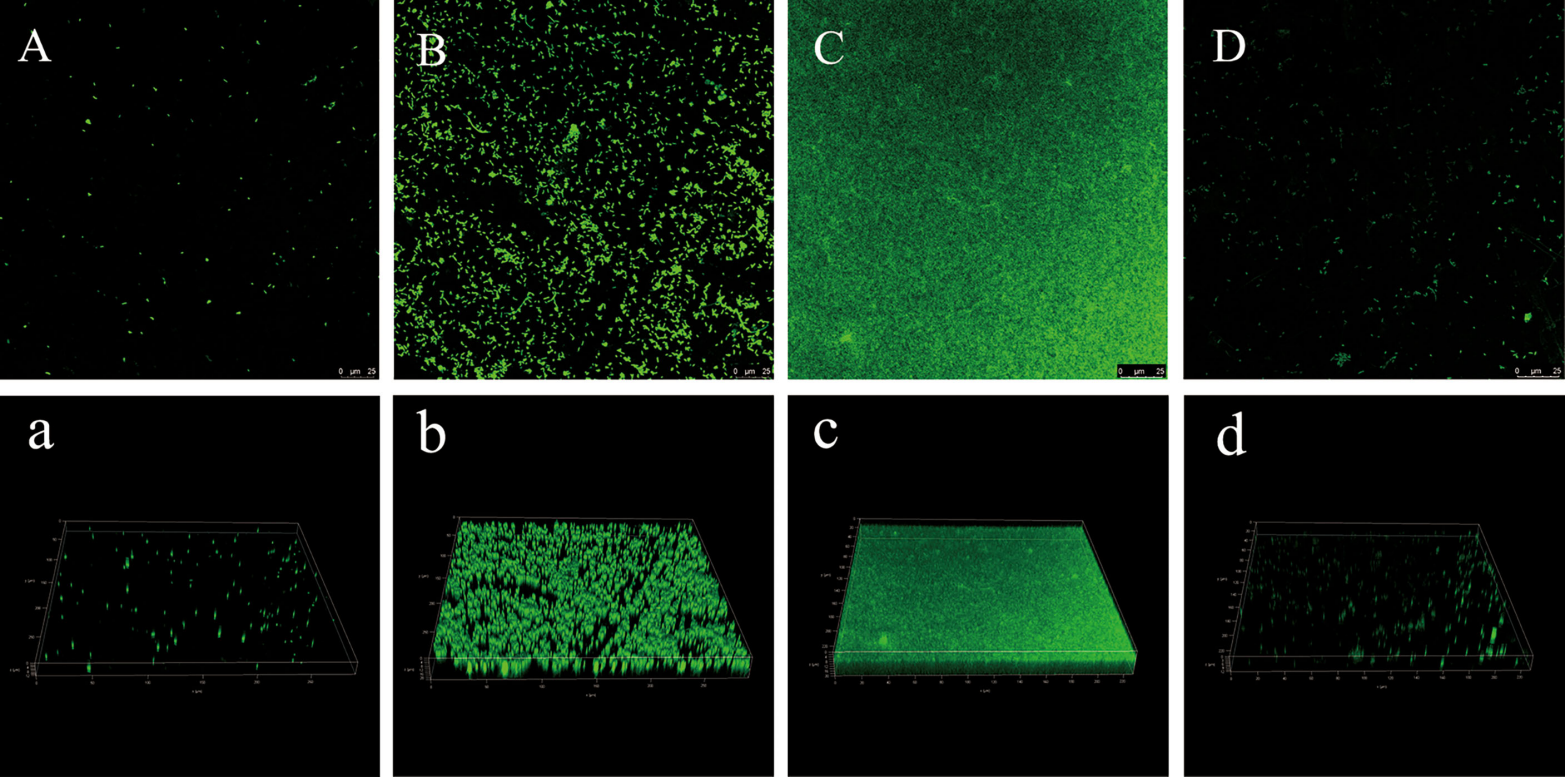
CLSM images of *V. parahaemolyticus* biofilm at different times. **(A/a)**: 6 h. **(B/b)**: 18 h. **(C/c)**: 36 h. **(D/d)**: 60 h.

In order to further observe the formation process of the biofilm, the three-dimensional structure of the biofilm at different times was observed more intuitively by CLSM (**Figure 5**). It can be observed from Fig. 4 that he fluorescence intensity of cells was weak at 6 h, showing a single-cell distribution (**Figure 5A/a**); at 18 h, the fluorescence intensity of cells became stronger, aggregated into clusters, and showed a slightly loose biofilm structure (**Figure 5B/b**); At 36 h, the fluorescence intensity of the cells reached the strongest, forming a thick biofilm covering the entire surface (**Figure 5C/c**); At 60 h, the biofilm was obviously dispersed, Only a few single clusters of cells remained (**Figure 5D/d**). Similar to the result of crystal violet staining, *V. parahaemolyticus* formed the most and densest biofilm at 36 h.

### Changes in the EPS of *Vibrio Parahaemolyticus* Biofilm

**Figure 6:**
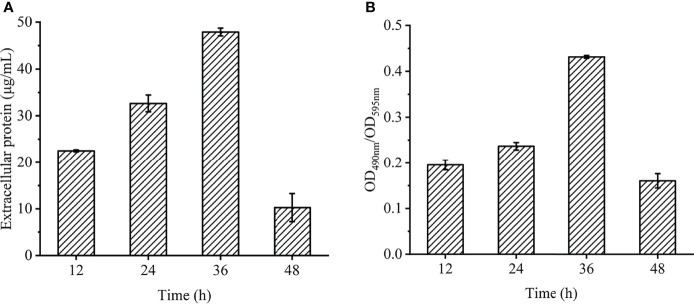
Chemical composition and contents of EPS in *V. parahaemolyticus* biofilm at different times. **(A)** extracellular protein. **(B)** extracellular polysaccharide.

On the basis of the above studies, the changes in the content of extracellular polymers in biofilms at different times were further explored (**Figure 6**). The amount of biofilm was related to the content of extracellular protein (**Figure 6A**) and extracellular polysaccharide (**Figure 6B**). The extracellular protein and polysaccharide gradually increased from 12 to 36 h, and reached the highest value at 36 h, which were 48.7 μL/mL and 0.43 (*OD*_490nm_/*OD*_595nm_), then began to decrease. Extracellular polysaccharide is an important component of biofilms. When bacteria initially adhere to the surface, they secrete viscous exopolysaccharides, which help themselves firmly adhere to the surface of objects and stimulate the aggregation of bacteria. All ectoproteins have a promoting effect on biofilm formation.

### Transcriptomic Comparison Between Planktonic and Biofilm Cells

**Figure 7:**
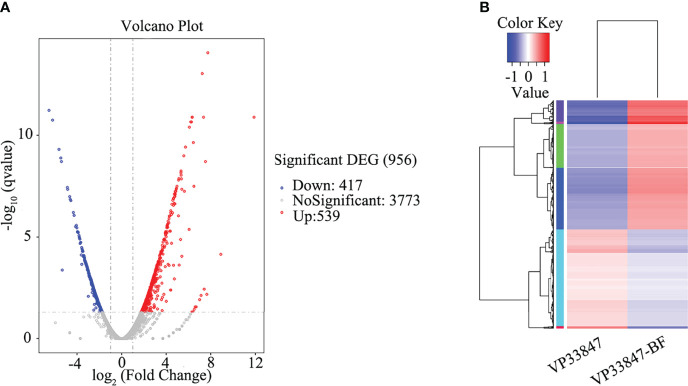
Differential expression analysis of experimental comparison group. **(A)** volcano plot. **(B)** hierarchical clustering plot.

Taking the planktonic bacteria (VP33847) as the control group and the biofilm (VP33847-BF) as the experimental group, a total of 956 differential genes were expressed, 537 genes were up-regulated and 417 genes were down-regulated (**Figure 7A**). Taking the FPKM value of the differential genes of VP33847 and VP33847-BF as the expression level, hierarchical clustering analysis was performed, and it was found that different culture conditions and environments caused the up-regulation and down-regulation of some genes involved in the same biological process, indicating that certain metabolic processes or cellular pathways played a decisive role in the formation of biofilm (**Figure 7B**). Through KEGG enrichment analysis, DEGs (differentially expressed genes) were enriched in TCS, ABC transporters environmental information processing pathway and QS cellular process pathway. There were 29 up- and 19 down-regulated DEGs in ABC transporters pathway, 25 up- and 23 down-regulated DEGs in TCS pathway and 12 up- and 15 down-regulated DEGs in QS pathway.

### Differential Expression Analysis of TCS-Related Genes

TCS is a signal transduction system that responds by sensing various changes in the external environment and its own state, and can sense nutritional status, external osmotic pressure and antibiotic concentration. *ompR* and *envZ* were upregulated by about 3.40 and 2.45, and their encoded proteins are the two-component system response regulator OmpR and the sensor histidine kinase EnvZ (**Table 1**). EnvZ/OmpR-TCS positively regulate *V. parahaemolyticus* biofilm formation. In response to external osmotic stress, EnvZ transmits a signal to OmpR with transcriptional activity by means of a phosphate group, thereby regulating the transcription of outer membrane porin (Wang et al., 2012) and participating in the regulation of biofilm formation. *cpxA* was upregulated by about 1.86, and its encoded protein is the envelope stress sensor histidine kinase CpxA (**Table 1**). The envelope pressure induced cell death (Snyder et al., 1995) and activated the envelope stress response of Cpx-TCS (Cosma et al., 1995). It can be seen that Cpx-TCS cooperates with EnvZ/OmpR-TCS to regulate *V. parahaemolyticus* biofilm formation.

**Table 1:** Analysis of differential gene expression of Two-component system.

GeneName	logFC	Product
*VP_RS20615*	7.63	cytochrome ubiquinol oxidase subunit I
*VP_RS22155*	5.10	phosphate ABC transporter substrate-binding protein
*VP_RS04430*	4.66	TRAP transporter substrate-binding protein
*VP_RS04435*	4.13	TRAP transporter small permease
*VP_RS15995*	3.98	response regulator transcription factor
*ompR*	3.40	two-component system response regulator OmpR
*VP_RS20550*	3.07	thiolase family protein
*VP_RS22100*	2.93	methyl-accepting chemotaxis protein
*VP_RS10510*	2.92	alkaline phosphatase
*VP_RS09250*	2.90	methyl-accepting chemotaxis protein
*VP_RS14060*	2.90	response regulator
*uhpT*	2.84	hexose-6-phosphate:phosphate antiporter
*envZ*	2.45	two-component system sensor histidine kinase EnvZ
*VP_RS12825*	2.37	methyl-accepting chemotaxis protein
*VP_RS22300*	2.15	methyl-accepting chemotaxis protein
*phoB*	2.09	phosphate regulon transcriptional regulator PhoB
*opaR*	2.08	transcriptional regulator OpaR
*VP_RS07205*	2.03	methyl-accepting chemotaxis protein
*VP_RS10975*	1.94	flagellin
*cpxA*	1.86	envelope stress sensor histidine kinase CpxA
*VP_RS00270*	1.84	nitrogen regulation protein NR(II)
*uvrY*	1.81	UvrY/SirA/GacA family response regulator transcription factor
*frdD*	-1.88	fumarate reductase subunit FrdD
*ccoP*	-2.05	cytochrome-c oxidase%2C cbb3-type subunit III
*VP_RS19140*	-2.31	sigma-54 dependent transcriptional regulator
*ccoO*	-2.32	cytochrome-c oxidase%2C cbb3-type subunit II
*VP_RS19865*	-2.32	anaerobic C4-dicarboxylate transporter
*VP_RS16160*	-2.33	sigma-54 dependent transcriptional regulator
*VP_RS09775*	-2.40	4Fe-4S dicluster domain-containing protein
*glnD*	-2.51	bifunctional uridylyl-transferase/uridylyl-removing protein GlnD
*VP_RS04450*	-2.52	sigma-54 dependent transcriptional regulator
*frdC*	-2.67	fumarate reductase subunit FrdC
*ttrA*	-2.80	tetrathionate reductase subunit TtrA
*frdA*	-2.81	fumarate reductase (quinol) flavoprotein subunit
*VP_RS13985*	-2.82	succinate dehydrogenase/fumarate reductase iron-sulfur subunit
*nrfD*	-2.87	polysulfide reductase NrfD
*VP_RS23430*	-3.39	sigma-54 dependent transcriptional regulator

*opaR* was upregulated by about 2.08 (**Table 1**). As a transcriptional regulator, *opaR* participated in TCS (Bramhachari et al., 2018), positively regulated the formation of biofilm and the expression of capsular polysaccharides (CPS), and affectd the colony morphology of biofilm, which was speculated to be related to pleated colonies.

*VP_RS19140*, *VP_RS16160*, *VP_RS04450* and *VP_RS23430* were downregulated by about 2.31, 2.33, 2.52 and 3.39, respectively, and their encoded proteins are all sigma-54 (σ^54^) dependent transcriptional regulators (**Table 1**). σ factor plays an important role in gene transcription and can combine with promoter elements to express an active RNA polymerase (Campbell et al., 2008). Among them, σ^54^ is mainly responsible for regulating genes related to environmental signals and requires additional energy to activate transcription. It can be seen that the external environment at 36 h is not enough to activate σ^54^ and has a negative regulatory relationship with the TCS of *V. parahaemolyticus*, but the specific mechanism is still unclear.

*VP_RS22100*, *VP_RS09250*, *VP_RS12825*, *VP_RS22300*, and *VP_RS07205* were upregulated by about 2.93, 2.90, 2.37, 2.15 and 2.03, respectively, and the encoded proteins are all methyl receptor chemotactic proteins (**Table 1**). Chemotaxis allows bacteria to tend to favor their own environmental factors in the face of environmental changes. Methyl-accepting chemotaxis protein (MCP) can cross the cell membrane and sense chemical changes in the environment, and then induces bacterial adaptation to growth by signaling itself (Porter et al., 2011). MCP was affected by histidine kinase and phosphorylated as a response protein. MCP had a positive regulatory relationship with the TCS of *V. parahaemolyticus*, and was simultaneously enriched for cell motility. *VP_RS10975* was upregulated by about 1.94, and its encoded protein is the flagellin FlgL. It is speculated that MCP interacts with flagellin to affect bacterial attachment and clustering.

*VP_RS04430* and *VP_RS04435* were upregulated by about 4.66 and 4.13, and their encoded proteins are TRAP transporter protein substrate binding protein and TRAP transporter protein small permease, which use ion electrochemical gradients to provide energy for solute uptake (Forward et al., 1997), indicating that the TRAP transporter system is not only involved in solute uptake but also cooperates with TCS to positively regulate the formation of biofilm.

### Differential Expression Analysis of QS-Related Genes

The QS system consists of signals and receptors, quorum sensing regulators, and other genes under control. The fold difference of *opaR* upregulated was about 2.08, and *luxS* was downregulated by about 2.32 (Guo et al., 2018) (**Table 2**). *opaR* is considered a major QS regulator involved in colony morphology, virulence and other biophysical aspects (Burke et al., 2015), and LuxS is a key enzyme in the LuxS/AI-2 QS system. *V. parahaemolyticus* biofilm formation was *opaR*-dependent at high cell density at 36 h. OpaR inhibited the production of biofilm, and the growth rate of biofilm was reduced to adapt to the environment and maintained the stability of bacterial growth.

**Table 2:** Analysis of differential gene expression of quorum sensing.

GeneName	logFC	Product
*oppB*	11.86	oligopeptide ABC transporter permease OppB
*VP_RS06525*	7.51	ABC transporter permease subunit
*VP_RS06535*	5.56	peptide ABC transporter substrate-binding protein
*VP_RS06515*	4.77	ATP-binding cassette domain-containing protein
*VP_RS06520*	4.32	ATP-binding cassette domain-containing protein
*VP_RS15290*	3.21	ABC transporter ATP-binding protein
*ribA*	2.91	GTP cyclohydrolase II
*VP_RS02600*	2.87	3-deoxy-7-phosphoheptulonate synthase
*lepB*	2.35	signal peptidase I
*VP_RS16920*	2.34	ABC transporter ATP-binding protein
*VP_RS20695*	2.30	ABC transporter ATP-binding protein
*opaR*	2.08	transcriptional regulator OpaR
*oppF*	-1.87	murein tripeptide/oligopeptide ABC transporter ATP binding protein OppF
*secB*	-1.90	protein-export chaperone SecB
*VP_RS00265*	-1.96	ABC transporter permease
*VP_RS10170*	-2.00	peptide ABC transporter substrate-binding protein
*VP_RS00270*	-2.01	ABC transporter permease
*VP_RS10155*	-2.13	ABC transporter ATP-binding protein
*VP_RS06490*	-2.27	ABC transporter permease
*VP_RS06495*	-2.30	ABC transporter permease
*luxS*	-2.32	S-ribosylhomocysteine lyase
*oppC*	-2.59	oligopeptide ABC transporter permease OppC
*VP_RS23465*	-2.63	peptide ABC transporter substrate-binding protein
*VP_RS06485*	-3.09	ABC transporter ATP-binding protein
*VP_RS00260*	-3.24	peptide ABC transporter substrate-binding protein

The differential fold of *oppB* up-regulation was about 11.86 (**Table 2**). *oppB*, *oppC*, and *oppF* are related to the transport of oligopeptides. The *oppB*-encoded protein is an oligopeptide ABC transporter permease (**Table 2**), which is an integral membrane protein and is highly hydrophobic. It is capable of mediating the passage of peptides across the cytoplasmic membrane, and is associated with resistance of biofilm (S.Masulis et al., 2020). Most of the QS-related DEGs were involved in ABC transporter, and their encoded proteins are mostly various binding proteins in ABC transporter and ABC transporter permease, indicating that QS can influence multiple ABC transporters to actively transport certain substrates into and out of cells to adapt to biofilm formation.

*VP_RS02600* was upregulated by about 2.87. The encoded protein is 3-deoxy-7-phosphogentanoate synthase (**Table 2**), which is associated with extracellular polysaccharides and extracellular proteins. The cells aggregate and then secrete extracellular polysaccharides and extracellular proteins, which firmly adhere to the surface of the object and play a decisive role in the strong adhesion of the biofilm. QS promoted the secretion of extracellular polysaccharides and extracellular proteins and was regulating the formation of the biofilm.

### COG Annotation Analysis of DEGs

The main Cluster of Orthologous Groups of proteins (COG) classifications of DEGs were Amino acid transport and metabolism, General function prediction only, Function unknown, Inorganic ion transport and metabolism, Cell motility, etc. Among the genes related to cell movement, in addition to flagellin significantly increased, MCP and CpxP family proteins were significantly increased, which confirmed the analysis results of TCS differential genes. MCP stimulates the autophosphorylation of histidine kinases, while CpxP, a related protein of CpxA, can inhibit the autophosphorylation of CpxA (Tschauner et al., 2014). Upregulation of *vscN* was also found in cell motility-related genes, and its encoded protein is the SctN family type III secretory system ATPase VscN, which is associated with *V. parahaemolyticus* virulence. The highly conserved T3SS protein is similar to that of the bacterial flagellar matrix (Aizawa, 2001), so it is thought that T3SS and bacterial flagella have similar expression during biological evolution, and that the flagellum is an adhesion of bacteria to the surface of host cells when VscN is involved in the pathogenesis of *V. parahaemolyticus* by facilitating bacterial contact with human cells and activating T3SS when *V. parahaemolyticus* comes into contact with the host cell (Zhuang et al., 2021).

## Discussion

**Figure 8:**
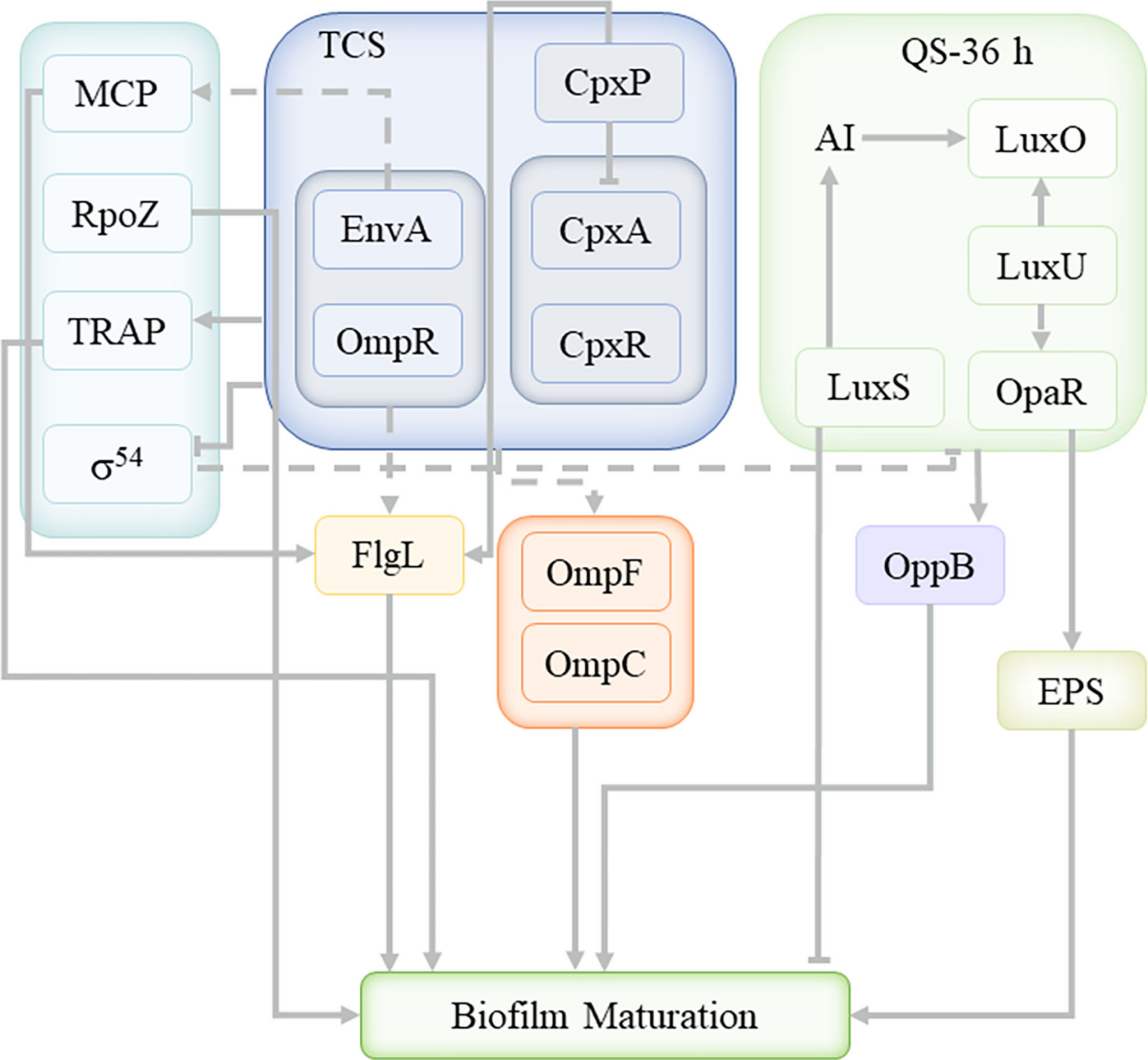
The transcriptional network of *V. parahaemolyticus* biofilm formation in 36 h.

In the present work, we successfully used Illumina RNA-sequencing to identify DEGs in the planktonic and biofilm cells. The data revealed that planktonic and biofilm cells had significantly different expression patterns (**Figure 8**), with 537 genes upregulated and 417 genes downregulated out of 956 DEGs. 48 DEGs were enriched to TCS and 27 DEGs were enriched to QS. Analysis of DEGs revealed that Cpx-TCS and EnvZ/OmpR-TCS positively regulate *V. parahaemolyticus* biofilm formation in response to envelope pressure and osmotic pressure. Siryaporn and Goulian studied the inhibition of crosstalk between CpxA/CpxR and EnvZ/OmpR two-component systems in *Escherichia coli*, and successfully discovered two mechanisms that suppress cross-talk between these two proteins (Siryaporn and Goulian, 2008); Batchelor et al. showed that Cpx-TCS and EnvZ/OmpR-TCS converge at the porin promoters to regulate the classical porins OmpF and OmpC (Batchelor et al., 2005). It was demonstrated that Cpx-TCS synergized with EnvZ/OmpR-TCS to promote biofilm formation, similar to the results of this study.

The *V. parahaemolyticus* TCS system positively regulated MCP and promoted biofilm formation by affecting the *V. parahaemolyticus* flagella. Minamino et al. found the phosphorylated form of the chemotactic signaling protein CheY binded to FliM and FliN for switching the direction of flagellar motor rotation (Minamino et al., 2019). Dons et al. studied role of the two-component CheA/CheY system of *Listeria monocytogenes* in swarming and tumbling behavior during motility (Dons et al., 2004). This suggestd that MCP was involved in the TCS system of *V. parahaemolyticus*, and affectd bacterial movement direction and surface attachment through flagellin, but the histidine kinase that activates the conformational change of MCP itself remains unclear.

σ^54^ factor-related genes were enriched and downregulated with TCS, indicating a negative regulatory relationship with TCS of *V. parahaemolyticus*. Yan et al. found that in *Pseudomonas fluorescens* 2P24, the sigma factor RpoS can only negatively regulate the quorum-sensing *pcoI* gene in the context of a functional GacS/GacA system (Yan et al., 2009). Thus, the σ^54^ factor appears to negatively regulate QS under specific TCS system conditions. *rpoZ* was not enriched in the TCS pathway, and was also an important sigma factor, upregulated by about 1.94, and its encoded protein is the DNA-directed RNA polymerase subunit ω. It was speculated that *rpoZ* played a promoting role in the process of *V. parahaemolyticus* biofilm maturation. Mukherjee and Chatterji found that *Mycobacterium smegmatis* lacking the *rpoZ* gene formed a mutant biofilm with defective maturation (Mukherjee and Chatterji, 2008), indicating that *rpoZ* plays a key role in the maturation of the biofilm.

At present, only a few physiological mechanisms of TRAP-T have been elucidated. David J. Kelly and Thomas found that DctPQM-T of *Rhodobacter capsulatus* used DctS/DctR-TCS to activate the *dctPQM* operon by sensing tetracarbonate in the periplasm (Kelly and Thomas, 2001). Morris et al. described that TRAP transporters are widely used in marine bacteria that live in Na^+^-rich environments and that are nutritionally poor (Morris et al., 2002). In the present work, it was confirmed that the TRAP transport system in *V. parahaemolyticus* cooperated with TCS to positively regulate the formation of biofilm, and the specific mechanism still needs further study.

As the main QS regulator of *V. parahaemolyticus*, OpaR was enriched in both TCS and QS, and upregulated, indicating that there is a positive regulatory relationship between QS and TCS systems. At 36 h, the QS of *V. parahaemolyticus* was OpaR-dependent, consistent with a high-density bacterial environment. At the same time, it interacted with the ABC transport system, similar to the conclusion drawn by Yuan et al. that the LuxS/AI-2 QS system mainly affected the transport, conversion and metabolism of ABC transporter proteins and carbohydrates (Yuan et al., 2021). Li et al. proposed that transcriptomic profiles of *Escherichia coli* (*E. coli*) treated with heavy ion (HI) illustrated that HI might perform membrane damage through regulating material transport, inhibited LuxS/AI-2 system, finally impeded biofilm formation (Li et al., 2021). The ABC transport system plays a key role in the formation of biofilms, and cooperates with QS to regulate the formation of biofilms, but the mechanism of interaction between QS and ABC transporter system needs to be further investigated. Li et al. suggested that the enhancement of AHL-based QS favored the EPS component productivity (Li et al., 2014). Hu et al. proposed that QS promoted the secretion of exopolysaccharides, regulating the formation and consolidation of biofilm (Hu et al., 2020). QS promoted the secretion of extracellular proteins and extracellular polysaccharides, which was consistent with the previous EPS analysis results. Extracellular proteins and extracellular polysaccharides promote the formation of biofilms. In the process of cell growth, in order to stabilize the growth of cells, various regulatory mechanisms simultaneously carry out different positive and negative regulation.

Studying the regulation mechanism related to biofilms makes the action process of the regulation mechanism more comprehensive, lays a foundation for further exploration of the specific mechanism, and is of great significance to the edible safety of aquatic products and the risk assessment of *V. parahaemolyticus*.

## Conclusion

During the mature stage of *V. parahaemolyticus* biofilm, TCS and QS cooperate to regulate the formation of biofilm, and there is a positive regulatory relationship between QS and TCS system. At this time, Cpx-TCS and EnvZ/OmpR-TCS responded to envelope pressure and osmotic pressure to and the TRAP transport system cooperated with TCS to positively regulate biofilm formation under the influence of environmental energy. In addition, MCP and RpoZ were enriched in the TCS system, which affect the flagella and biofilm maturation, respectively; while the σ^54^ factor was inhibited by environmental factors. At the same time, QS interacted with the ABC transport system to jointly promote the secretion of extracellular proteins and exopolysaccharides, which contribute to the formation of biofilms.

## Data Availability Statement

The data presented in the study are deposited in the NCBI repository, accession number PRJNA827017.

## Author Contributions

QW and JO designed the experiments, analysed the data and drafted the manuscript. QW, PL and PW carried out the experiment. JO conceptualized the idea and edited the manuscript. All authors reviewed the results and approved the final version of the manuscript.

## Funding

This research was funded by Innovation Program of Shanghai Municipal Education Commission (2017-01-07-00-10-E00056).

## Conflict of Interest

The authors declare that the research was conducted in the absence of any commercial or financial relationships that could be construed as a potential conflict of interest.

## Publisher’s Note

All claims expressed in this article are solely those of the authors and do not necessarily represent those of their affiliated organizations, or those of the publisher, the editors and the reviewers. Any product that may be evaluated in this article, or claim that may be made by its manufacturer, is not guaranteed or endorsed by the publisher.
